# Diet and The Risk of Endometriosis in Iranian Women: A Case-Control Study

**DOI:** 10.22074/ijfs.2020.44378

**Published:** 2020-10-12

**Authors:** Mahnaz Ashrafi, Nadia Jahangiri, Shahideh Jahanian Sadatmahalleh, Fatemeh Aliani, Mohammadreza Akhoond

**Affiliations:** 1 Department of Endocrinology and Female Infertility, Reproductive Biomedicine Research Centre, Royan Institute for Repro- ductive Biomedicine, ACECR, Tehran, Iran; 2 Department of Obstetrics and Gynaecology, Faculty of Medicine, Iran University of Medical Sciences, Tehran, Iran; 3 Department of Reproductive Health and Midwifery, Faculty of Medical Sciences, Tarbiat Modares University, Tehran, Iran; 4 Department of Obstetrics and Gynaecology, Faculty of Medicine, Tehran University of Medical Sciences, Tehran, Iran; 5 Department of Statistics, Mathematical Sciences and Computer Faculty, Shahid Chamran University of Ahvaz, Ahvaz, Iran

**Keywords:** Diet, Endometriosis, Risk

## Abstract

**Background:**

Endometriosis is one of the most common pelvic diseases associated with dyspareunia, pelvic pain, and infertility. The primary aim of this study is to evaluate the role of diet on the risk of endometriosis among Iranian women.

**Materials and Methods:**

This case-control study was conducted in two health research centres between 2015 and 2016. There were 207 women with endometriosis (case) and 206 women without endometriosis (control) who were evaluated by laparoscopy. The women were asked about their frequency of consumption per week of portions of se- lected dietary items in the Iranian diet in the year before the interview.

**Results:**

The results indicated that intake of green vegetables (odds ratio [OR]=0.39, 95% confidence interval [CI]=0.21–0.74, Ptrend=0.004), red meat (OR=0.61, 95% CI=0.41–0.91, Ptrend=0.015) and dairy products (milk [OR=0.65, 95% CI=0.47–0.92, Ptrend=0.014], cheese [OR=0.53, 95% CI=0.37–0.76, Ptrend<0.001]), fresh fruit (OR=0.68, 95% CI=0.50–0.93, Ptrend=0.015) and grain legumes (OR=0.59, 95% CI=0.47–0.77; Ptrend<0.001) had a significant association with lower risk of endometriosis. Consumption of carrots, green tea, fish, eggs and oil was not significantly related to the risk of endometriosis.

**Conclusion:**

This study suggests that certain types of dietary components may be related to the risk of endometriosis.

## Introduction

Endometriosis is one of the most common hormonedependent gynaecological diseases. It is a chronic inflammatory gynaecological condition that is characterized by dyspareunia, pelvic pain, and infertility (1). Despite the high association with morbidity, the prevalence, incidence, aetiology and risk factors of endometriosis remain elusive (2). Endometriosis may be related to genetic, hormonal, anatomic, immune, inflammatory, environmental and lifestyle factors (3). The role of diet in several chronic diseases has been determined (4). Oestrogen activity is a common denominator for many known endometriosis risk factors and an association has been demonstrated between diet and oestrogen dependency; therefore, endometriosis may also be affected by diet (1, 5). The role of diet in the incidence and progression of endometriosis has become a growing field of interest in recent years (6, 7) and summarized in a recent review (8). Diet may play a role in its etio-pathogenesis (9), and can be influenced through multiple pathways that include effects on oxidative stress, prostaglandin (PG) metabolism, smooth muscle contractility, inflammation, immune function and estrogenic effects (10). Huang et al. (11) reported that dietary factors altered serum sexhormone concentrations and activity. A few human studies explored the relationship between diet and endometriosis risk, but had conflicting results (1, 2, 12). Parazzini et al. (12) found no association between the consumption of milk, cheese, carrots, fish and whole grain foods and the risk of endometriosis; however, there was a significant association between the intake of green vegetables, fresh fruit, and red meat with endometriosis risk. Alternatively, Yamamoto et al. (7), in a large cohort study, reported a significant association between the consumption of red meat and the risk of endometriosis, while consumption of fish, poultry, and eggs were unrelated to endometriosis risk. Another large US cohort study revealed that total fat consumption was not associated with the risk of endometriosis (2). Of note, most published studies supported the association used animal models of endometriosis and extrapolated the results to humans (13). According to the limited number of studies on this topic and inconsistencies between these prior study findings, the present analytic research was conducted to further investigate if the selected diet factors affect the occurrence of endometriosis in Iranian women.

## Materials and Methods

### Study Population

This case-control study was carried out at two referral centres, Royan Institute and Vali-Asr Reproductive Health Research Centre (both in Tehran, Iran), between April 2015 and March 2016. The Institutional Review Board and the Medical Ethics Committee of Tehran University of Medical Sciences approved this study. All procedures performed in this study were in accordance with the Declaration of Helsinki and an informed consent was obtained from all individual participants included in the study.

The control and case groups were from both centres. A total of 510 women who underwent diagnostic laparoscopy were recruited for this study. The main indications for laparoscopy were symptoms of endometriosis (dysmenorrhea, dyspareunia and pelvic pain), uterine abnormality, tuboperitoneal disorder and unexplained factor in infertility. Women (n=97) who had adhesions, leiomyomas, fibromas, and/or uterine abnormalities at laparoscopy were excluded from the study. Finally, we included 413 women who were divided into two groups according to the laparoscopy findings of endometriosis (case group) or normal pelvis (control group). Following surgery, the stage of the disease was defined according to the classification system of the revised American Society for Reproductive Medicine (rASRM) as stage I (minimal), stage II (mild), stage III (moderate) and stage IV (severe) (14). Histologic confirmation was obtained in 79.6% of the women with endometriosis.

### Dietary Assessment

The required data were collected using a structured questionnaire for information on demographic variables and reproductive characteristics. For each selected dietary item in the Iranian diet, the participants were asked about their frequency of consumption per week (i.e., 14 meals) in the year before the interview. The questionnaire was a structured questionnaire similar to that reported by Parazzini et al. in their study (12) on diet habit; specifically, the selected dietary items were green vegetables (0-6, 7-12≥13 portions/ week), fresh fruits (≤6, 7-13≥14 portions/week), carrots (0, 1≥2 portions/week), grain legumes (0, 1≥2 portions/ week), red meat (0-3, 4-6≥7 portions/week), fish (0, 1≥2 portions/week), milk (0, 0.5-6≥7 portions/week), cheese (≤2, 3-5≥6 portions/week), eggs (0, 1≥2 per week) and green tea (yes or no). The items of green vegetables and fruits included all types, specifically all of the main sources in the Iranian diet such as spinach/other greens, kale, green salads, broccoli, cauliflower, citrus fruit, apples, peaches, melons, strawberries/cherries, bananas and pears.

In terms of content validity, we requested that 10 experts in the fields of nutrition, midwifery, reproductive health and gynaecology review the questionnaire and assess each item based on four criteria: relevancy, clarity, simplicity, and necessity. The content validity ratio (CVR) was calculated based on the responses to the necessity of questions (nE) according to the following formula.

CVR= (nE-N/2)/ (N/2)

Lawshe’s table was used to determine the CVR cut-off point (15). According to Lawshe, for 10 professionals, the minimum required CVR for each item is 0.94. The content validity index (CVI) for this questionnaire was based on the Waltz and Bausell CVI (16). The CVI for each item was obtained by dividing the number of professionals who ranked the items as compatible or full compatible for each criterion (relevancy, clarity, simplicity, and necessity) to the total number of professionals. The average value of the three criteria was used as the total CVI for each item. The minimal required amount of CVI for each item was 0.90 (17). A testretest analysis with an interval of 15 days was approved in a pilot study of 30 women with endometriosis. We assessed the test-retest reliability of the questionnaire by using two correlational measures, Spearman’s correlation and Cohen’s kappa, to show the similarity in the responses to an item on test and retest (18). According to Field (18) and Cade et al. (19) large correlation coefficients of 0.5 or greater indicate high reliability. The value of Kappa identifies the strength of the agreement according to the categories reported by Masson et al. (20) of poor (<0.20), fair (0.21-0.40), moderate (0.41-0.60), good (0.61-0.80), and very good (0.81-1.00). Table 1 shows that all correlation coefficients are above 0.6, which indicates good reliability.

**Table 1:** Spearman’s r and Cohen’s kappa correlational measures between test and retest


Food item consumption	Spearman’s r ^b^	Cohen’s kapp^a^

Green vegetables	0.768	0.627
Fresh fruits	0.960	0.880
Carrots	0.873	0.827
Grain legumes	0.847	0.759
Green tea	0.768	0.627
Type of oi1^a^	-	0.655
Fish	0.967	0.948
Eggs	0.606	0.663
Meat	0.789	0.701
Milk	0.703	0.660
Cheese	0.818	0.748


^a^; Mode in test and retest is reported, ^b^; Because this is a nominal variable, and Cramer's V correlation coefficient was used.

### Statistical analysis

The sample size was calculated according to the studied variables and based on the “rule of thumb” method. We considered at least 10 samples per variable; therefore, the sample size was estimated to be 200 women (21). The chi square or t-tests, when appropriate, were employed using SPSS software (version 22, USA) to compare categorical and continuous variables, respectively. We used logistic regression to determine the risk factors associated with endometriosis. In addition, logistic regression was used to adjust for age, education levels and body mass index (BMI). The odds ratio (OR) and 95% confidence interval (CI) were outputted for each of the calculated factors. In order to test the linear trend for ordinal variables such as green vegetables, fresh fruits, carrots, grains legumes, fish, eggs, meat, milk and cheese, we reported the OR and P value for the trend using category medians. P values less than 0.05 were considered to be statistically significant.

## Results

The distribution of cases and controls according to age and selected characteristics is presented in Table 2. The severity of disease was staged according to the rASRM classification of endometriosis. Endometriosis was staged as minimal (rASRM stage I) in 56 (27.1%), mild (rASRM stage II) in 24 (11.6%), moderate (rASRM stage III) in 44 (21.3%), and severe (rASRM stage IV) in 83 (40.1%). When the demographic characteristics of the women with endometriosis were compared with the control group, there were no significant differences detected in the age at menarche, parity, occupation, cigarette smoking and history of infertility. In contrast, there was a significant difference concerning age, education and BMI between the two groups. The mean age was 31.50 ± 5.52 years in the endometriosis group and 30.30 ± 5.96 years in the control group (P=0.02).

**Table 2:** Comparison of demographic characteristics of endometriosis cases and controls.


Parameters	Control N (%)	Case N (%)	P Value^*^

Age (Y)			
≤24	34 (16.5)	17 (8.2)	0.02
25-34	127 (61.7)	131 (63.3)	
≥35	45 (21.8)	59 (28.5)	
Age at menarche (Y)			
<12	17 (8.3)	11 (5.3)	0.47
12	53 (25.7)	58 (28.0)	
>12	136 (66.0)	138 (66.7)	
Parity			
0	160 (77.7)	154 (74.4)	0.54
1	40 (19.4)	43 (20.8)	
≥2	6 (2.9)	10 (4.8)	
Education levels			
Under diploma	76 (36.9)	45 (21.7)	0.004
Completed high school	74 (35.9)	91 (44)	
University	56 (27.2)	71 (34.3)	
Occupation			
Housewife	170 (82.5)	157 (75.8)	0.09
Employed	36 (17.5)	50 (24.2)	
BMI (kg/m^2^)			
<20	7 (3.4)	20 (9.7)	0.001
20-24.9	68 (33.0)	92 (44.4)	
25-29.9	93 (45.1)	74 (35.7)	
≥30	38 (18.4)	21 (10.1)	
Smoking			
No	202 (98.1)	204 (98.6)	0.69
Yes	4 (1.9)	3 (1.4)	
History of infertility			
Yes	182 (88.3)	182 (87.9)	0.89
No	24 (11.7)	25 (12.1)	


^*^ Chi square test, BMI: Body mass index, and Y; Year.

Table 3 shows the relationship between selected food intake and the risk of endometriosis. Our results indicated that intake of green vegetables (Ptrend=0.004) and red meat (Ptrend=0.015) were significantly associated with a lower risk for endometriosis. Fresh fruits (Ptrend=0.015), dairy (milk [Ptrend=0.014] and cheese [Ptrend<0.001]), and grain legumes (Ptrend<0.001) were associated with a decreased risk for endometriosis. Table 3 shows an OR greater than one for those with zero or one portion of grain legumes per week compared to those with two portions of grain legumes per week. Decreased grain legume intake appears to be a risk factor whereas higher grain legume intake appears to be protective. Consumption of carrots, green tea, fish, eggs, and oil were not significantly related to the risk for endometriosis.

**Table 3:** Risk of endometriosis and selected food intake


Food item consumption No. of servings	Control N (%)	Case N (%)	OR unadjusted (95% CI)^a^	OR adjusted (95% CI)^a^	P Value*

Green vegetables					0.03
≤6 portions/week	176 (85.4)	193 (93.2)	1†	1†	
7-12 portions/week	25 (12.1)	13 (6.3)	0.47 (0.23-0.95)	0.47 (0.22-1.02)	
≥13 portions/week	5 (2.4)	1 (0.5)	0.18 (0.02-1.57)	0.13 (0.01-1.21)	
P value for trend^**^	-	-	0.45 (0.25-0.82)	0.39 (0.21-0.74)	0.004
Fresh fruits					0.04
≤6 portions/week	127 (61.7)	143 (69.1)	1†	1†	
7-12 portions/week	43 (20.9)	47 (22.7)	0.97 (0.60-1.56)	0.92 (0.54-1.58)	
≥13 portions/week	36 (17.5)	17 (8.2)	0.41 (0.22-0.78)	0.43 (0.22-0.85)	
P value for trend^**^	-	-	0.71 (0.54-0.94)	0.68 (0.50-0.93)	0.015
Carrots					0.40
0 portions/week	93 (45.1)	77 (37.2)	1†	1†	
1 portions/week	55 (26.7)	57 (27.5)	1.25 (0.77-2.01)	1.25 (0.75-2.07)	
≥2 portions/week	58 (28.2)	73 (35.3)	1.52 (0.96-2.40)	1.38 (0.84-2.27)	
P value for trend^**^	-	-	1.23 (0.98-1.55)	1.16 (0.91-1.47)	0.232
Grain legumes					<0.001
0 portions/week	76 (36.9)	89 (43.0)	1†	1†	
1 portions/week	40 (19.4)	75 (36.2)	1.60 (0.98-2.61)	1.54 (0.91-2.63)	
≥2 portions/week	90 (43.7)	43 (20.8)	0.40 (0.25-0.65)	0.32 (0.19-0.54)	
P value for trend^**^	-	-	0.66 (0.52–0.84)	0.59 (0.47–0.77)	<0.001
Green tea					0.51
Yes	47 (22.8)	50 (24.2)	1†	1†	
No	159 (77.2)	157 (75.8)	1.07 (0.68-1.69)	1.17 (0.71-1.93)	
P value for trend^**^	-	-	1.07 (0.68-1.69)	1.01 (0.63-1.65)	0.951
Type of oi1					0.32
Animal	6 (2.9)	3 (1.4)	1†	1†	
Vegetable	185 (89.8)	182 (87.9)	1.96 (0.48-7.98)	2.15 (0.47-9.80)	
Animal and vegetable	15 (7.3)	22 (10.6)	2.93 (0.63-13.59)	3.42 (0.65-17.82)	
Fish					0.49
0 portions/week	118 (57.3)	122 (58.9)	1†	1†	
1 portions/week	59 (28.6)	63 (30.4)	1.03 (0.66-1.59)	0.97 (0.61-1.56)	
≥2 portions/week	29 (14.1)	22 (10.6)	0.73 (0.39-1.34)	0.67 (0.35-1.30)	
P value for trend^**^	-	-	0.90 (0.68-1.18)	0.85 (0.63-1.14)	0.284
Eggs					0.07
0 portions/week	45 (21.8)	37 (17.9)	1†	1†	
1 portions/week	36 (17.5)	47 (22.7)	1.58 (0.85-2.93)	1.80 (0.94-3.47)	
≥2 portions/week	125 (60.7)	123 (59.4)	1.19 (0.72-1.97)	1.21 (0.71-2.06)	
P value for trend^**^	-	-	1.04 (0.82-1.33)	1.04 (0.81-1.35)	0.734
Meat					0.03
0-3 portions/week	144 (69.9)	166 (80.2)	1†	1†	
4-6 portions/week	50 (24.3)	35 (16.9)	0.60 (0.37-0.98)	0.53 (0.31-0.90)	
≥7 portions/week	12 (5.8)	6 (2.9)	0.43 (0.15-1.18)	0.47 (0.15-1.38)	
P value for trend^**^	-	-	0.63 (0.44-0.91)	0.61 (0.41-0.91)	0.015
Milk					0.04
<1 portions/week	117 (56.8)	141 (68.1)	1†	1†	
1-6 portions/week	33.0	57 (27.5)	0.69 (0.45-1.06)	0.67 (0.42-1.06)	
≥7 portions/week	21 (10.2)	9 (4.3)	0.35 (0.15-0.80)	0.39 (0.16-0.93)	
P value for trend^**^	-	-	0.64 (0.47-0.88)	0.65 (0.47-0.92)	0.014
Cheese					<0.001
≤2 portions/week	74 (35.9)	118 (57)	1†	1†	
3-5 portions/week	120 (58.3)	79 (38.2)	0.41 (0.27-0.62)	0.39 (0.25-0.61)	<0.001
≥6 portions/week	12 (5.8)	10 (4.8)	0.52 (0.21-1.27)	0.52 (0.21-1.33)	<0.001
P value for trend^**^	-	-	0.52 (0.37-0.73)	0.53 (0.37-0.76)	


^†^; Reference category, ^*^; P values are adjusted for age, education levels and body mass index (BMI), ^**^; Determined using category medians, ^a^ OR; Odds ratio, and CI; Confidence interval.

## Discussion

### Selected Dietary Items

#### Vegetables/Fruits

The findings of the present study indicate that higher intake of green vegetables (OR=0.39, 95% CI=0.21–0.74; Ptrend=0.004) and fresh fruits (OR=0.68, 95% CI=0.50–0.93; Ptrend=0.015) can lower the risk of endometriosis. In three investigations, the relationship between servings per week or day of fresh fruit and green vegetable intake and endometriosis risk were evaluated (15, 16, 27). Similar to our study, Parazzini et al. (12) reported that intake of fresh fruits and green vegetables decreased the endometriosis risk. In contrast, Trabert et al. (1) found that vegetable consumption was not related to risk of endometriosis; however, higher disease risk was associated with increased fruit intake. The study’s authors posited that the results could be associated with a higher percentage of pesticide consumption in the cultivation of fruit, which might produce reactive oxygen species and decrease the antioxidant capacity of fruits and vegetables. The use of organochlorine pesticides in fruits should not prohibit their use. Rather, the use of organic fruits or removing the peels from contaminated fruits should be considered (22). Harris et al. (23) prospectively assessed data collected from 70 835 premenopausal women and found that fruit intake was associated with a decreased endometriosis risk; among these, citrus fruits had strongest association and it was suggested that the presence of beta-cryptoxanthin in these foods was probably responsible for this phenomenon. Unlike our finding in green vegetables, they reported that consumption of some vegetables such as cruciferous vegetables, corn and peas or lima beans were related to a higher risk of endometriosis. This positive association was observed among women with no history of infertility, which might explain the inconsistency between our results and those reported by Harris et al. (23), given that the highly selected population (almost 90%) in our study consisted of infertile women.

Populations on vegetarian diets usually have elevated sex-hormone binding globulin (SHBG) levels (24). A low-fat diet also decreases the levels of oestrogen in both pre-menopausal and post-menopausal women (25). Oestrogen conjugates enter the hepatic circulation through the bile, and are interrupted by dietary fibre; this encourages faecal oestrogen elimination (26). Increased SHBG or reduced serum levels of oestrogen can decrease oestrogenic stimulation of the endometrium, and restrict the proliferation of tissues that produce PGs (12). Hormonal agents are a potential connection between endometriosis and diet, as unopposed oestrogens can increase endometriosis risk. More difficult to explain in biological terms is the protective effect of a fruit and green vegetable-rich diet. High levels of carotenoids, folic acid, vitamin C and lycopene in a diet rich in fruits and green vegetables may cause inhibition of cell proliferation (27). In addition, it seems that dietary fruits and green vegetables may be surrogates for fibre. Fibre, as mentioned previously (28), decreases enterohepatic circulation and may thereby decrease the risk or severity of endometriosis.

#### Red Meat/ Fish

The findings in this study were inconsistent with the results of many similar studies in other countries (1, 7, 12, 13, 29). Our results showed a decreased endometriosis risk for those with 4-6 portions of meat per weak compared to those with 0-3 portions of meat per week (OR=0.61, 95% CI=0.41–0.91; Ptrend=0.015). Four studies analysed the risk of endometriosis with frequent red meat consumption (1, 12, 13, 29, 30), which is a rich source of saturated fat. In an Italian case–control study (12) and a prospective cohort study (7), high intake of red meat increased the risk for endometriosis. In a Belgian matched case-control study with prospective recruitment, meat consumption was not linked with the risk of peritoneal endometriosis (29). In another case-control study (1), no association was found between the risk of endometriosis and increased servings of red meat. The effect of red meat consumption reported by Parazzini et al. (12) could be connected to the fat content and type of fat in meat. Meat diet contains large amounts of fat, which can further increase oestrogen levels (7), and is comparably higher in omega-6 fatty acids (FAs), which stimulates the production of proinflammatory PGs (31). Fung et al. (32) have reported that a high intake of meat is associated with elevated serum concentrations of oestrogen sulphate and oestradiol; consequently, its consumption might directly contribute to increased levels of circulating steroid hormone (33) and to the maintenance of the disease. This is an arguable topic in spite of comparable serum levels of oestradiol in women with and without endometriosis (34), although the likelihood of elevations in local oestrogen synthesis with increased red meat consumption cannot be excluded (13). In a most recent study, Yamamoto, et al. (7) demonstrated that the effect of high intake of meat among endometriosis women with no report of infertility was partly related to the relationship between heme iron intake and endometriosis, with the mechanism of inflammation triggered by oxidative stress which involved in pathophysiology of the endometriosis.

There are several possible explanations for the inconsistency between our results and those obtained by other studies. One of the reasons for this discrepancy could be the kind of meat consumed in Iran and other countries. Beef and lamb are the most widely consumed meats in Iran, whereas in most other countries, pork is one of the highly consumed meats. Dioxin contamination in food products and animal food in Italy that occurred during the entire study is another factor that could impact the study results. The method for cooking meat is one of the most effective factors that varies in different nations and cultures (35). Another possible explanation for this might be the different slaughter procedures for sheep and calves in Iran (ritual cutting) and other countries (captive bolt stunning). Schulze et al. (36) explained that in ritual cutting, animals suffer much less pain and less stress hormones. Also, as the heart of animals killed by this way works much later, more blood and other materials, including hormones, will be removed from the animals (36); this could possibly be corroborated by other studies in which indicated an association between heme iron intake and endometriosis risk (7) and also reported lower concentrations of hemopexin, which is the major vehicle for the transportation of heme iron (37). In the present study, we found no association between the consumption of fish and endometriosis risk. This finding was consistent with the results of similar studies conducted by Parazzini et al. (Italy), Trabert et al. (Belgium) and Heilier et al. (USA) (1, 12, 29). Harel et al. (38) reported that fish consumption had the potential to reduce PGE2 and PGF2α concentrations and could be a possible risk-reducing factor for endometriosis.

#### Dairy products

Our findings suggest that dairy product consumption was associated with a reduced risk of endometriosis. As higher intake of milk (OR=0.65, 95% CI=0.47–0.92; Ptrend=0.014) and intake of 3-5 portions of cheese (OR=0.53, 95% CI=0.37-0.76; Ptrend<0.001)] was associated with a decreased risk of endometriosis. Few studies have examined the association between the intake of dairy foods and nutrients with risk of endometriosis. In the first human study that evaluated intake of dairy, Parazzini et al. (12) reported no association between milk or cheese consumption and risk of endometriosis. Alternatively, Trabert et al. (1) found a non-significant inverse correlation between dairy intake and risk of endometriosis. The results of a prospective cohort study revealed that high consumption of dairy products, specifically yogurt and ice cream during adolescence, was associated with a lower risk of endometriosis (6). It has been shown that serum and peritoneal fluid proinflammatory cytokine concentrations are elevated in women with endometriosis (8). Dairy products may be related to the endometriosis-associated inflammatory responses (8, 10). Zemel et al. (39) stated that a milk diet decreased inflammatory markers and oxidative stress, including interleukin-6 (IL-6) and tumour necrosis factoralpha receptor 2 (TNF-α R2). Another hypothesis for this association is the ability of calcium and vitamin D to down-regulate insulin-like growth factor-I (IGF-1), which plays a role in the growth-promoting process and up-regulation of transforming growth factor beta (TGF-β) acts as a negative autocrine growth factor (1).

#### Grains

Our result shows an OR lower than one for those with ≥ 2 portions (OR=0.59, 95% CI=0.47-0.77; Ptrend<0.001) of grain legumes per week compared to the reference group. Higher grain legumes intake appears to be protective. An analysis of the number of servings per week of grain did not show any association with the risk of endometriosis according to Trabert et al. (1). Similarly, no association was reported between the intake of grain and endometriosis risk (12, 30). Refined cereals can influence glycaemic load (GL) and glycaemic index (GI). These variables are used to estimate the rate of carbohydrate absorption and subsequent insulin demand. When insulin binds to its receptor in the endometrium, it is able to induce the growth of endometrial stromal cells. Moreover, hyperinsulinaemia increases the level of oestrogens through reducing the serum level of SHBG and increases the level of IGF-1 by lowering the serum level of insulin-like growth factor-binding protein 1 (IGFBP-1). Both IGF-1 and oestrogens stimulate endometrial cell proliferation (31). Accordingly, cereal consumption could be correlated with endometriosis risk.

Our data do not support an association between endometriosis risk and intake of any of the other nutrients or food groups evaluated in this study (e.g., carrots, green tea, oil, and eggs).

In the present results, oil intake was not associated with endometriosis risk (p-trend=0.32); this finding was similar to our previous study (30) performed on 156 infertile patients (p-trend=0.21). In the Italian case–control study, no association was found between oil consumption and risk of endometriosis (12).

This study has some limitations. An inevitable limitation is that case-control studies in nutritional epidemiology may be at a potential risk for recall bias. Information depends entirely on memory and there may be possible variations in recall bias for cases versus controls. Cases may associate their disease to their bad dietary habits and may over-report consumption of foods considered unhealthy. However, we believe that since the majority of women interviewed were probably unaware of the possible relationship between diet and endometriosis, the effect of recall bias was relatively low. As approximately 90% of our study population in both groups were infertile, this might limit the generalisability of results to all endometriosis women. Thus, our findings have implications for women with endometriosis from infertility clinic-based studies. Another weak point of our study was that we did not use the food frequency questionnaire (FFQ) due to the disadvantages of longer food lists and an additional respondent burden (40). The strong point of our study was the detailed availability of all records for the 413 participants. All of the participants completed the questionnaire.

## Conclusion

Despite the limitations, this research demonstrates that there is some association between intake of green vegetables, red meat, dairy products, cheese, fresh fruit and grain legumes with lower risk of endometriosis. These results highlight the necessity for appropriate extensive prospective evaluations to study these factors in fertile women with endometriosis to increase generalization of the findings.

## References

[1] Trabert B, Peters U, De Roos AJ, Scholes D, Holt VL (2011). Diet and risk of endometriosis in a population-based case-control study. Br J Nutr.

[2] Missmer SA, Chavarro JE, Malspeis S, Bertone-Johnson ER, Hornstein MD, Spiegelman D (2010). A prospective study of dietary fat consumption and endometriosis risk. Hum Reprod.

[3] Parazzini F, Esposito G, Tozzi L, Noli S, Bianchi S (2017). Epidemiology of endometriosis and its comorbidities. European Journal of Obstetrics & Gynecology and Reproductive Biology.

[4] Harvie M, Howell A, Evans DG (2015). Can diet and lifestyle prevent breast cancer: what is the evidence?.Am Soc Clin Oncol Educ Book.

[5] Jurkiewicz-Przondziono J, Lemm M, Kwiatkowska-Pamula A, Ziolko E, Wojtowicz MK (2017). Influence of diet on the risk of developing endometriosis. Ginekol Pol.

[6] Nodler JL, Harris HR, Chavarro JE, Frazier AL, Missmer SA (2020). Dairy consumption during adolescence and endometriosis risk.Am J Obstet Gynecol.

[7] Yamamoto A, Harris HR, Vitonis AF, Chavarro JE, Missmer SA (2018). A prospective cohort study of meat and fish consumption and endometriosis risk. Am J Obstet Gynecol.

[8] Saguyod SJU, Kelley AS, Velarde MC, Simmen RC (2018). Diet and endometriosis-revisiting the linkages to inflammation. J Endometr Pelvic Pain Disord.

[9] Parazzini F, Vigano P, Candiani M, Fedele L (2013). Diet and endometriosis risk: a literature review. Reprod Biomed Online.

[10] Harris HR, Chavarro JE, Malspeis S, Willett WC, Missmer SA (2013). Dairy-food, calcium, magnesium, and vitamin D intake and endometriosis: a prospective cohort study. Am J Epidemiol.

[11] Huang M, Liu J, Lin X, Goto A, Song Y, Tinker LF Relation of dietary carbohydrates intake to circulating sex hormone-binding globulin levels in postmenopausal women.J Diabetes.

[12] Parazzini F, Chiaffarino F, Surace M, Chatenoud L, Cipriani S, Chiantera V (2004). Selected food intake and risk of endometriosis. Hum Reprod.

[13] Simmen RC, Kelley AS (2018). Seeing red: diet and endometriosis risk. Ann Transl Med.

[14] Revised American Fertility Society classification of endometriosis (1985). 1985. Fertil Steril.

[15] Msyamboza KP, Ngwira B, Dzowela T, Mvula C, Kathyola D, Harries AD (2011). The burden of selected chronic non-communicable diseases and their risk factors in Malawi: nationwide STEPS survey. PLoS One.

[16] Waltz CF, Bausell BR (1981). Nursing research: design statistics and computer analysis.

[17] Kellar SP, Kelvin EA (2012). Munro's statistical methods for health care research: 6th ed.Philadelphia.Lippincott Williams & Wilkins.

[18] Field A (2013). Discovering statistics using IBM SPSS statistics.

[19] Cade J, Thompson R, Burley V, Warm D (2002). Development, validation and utilization of food-frequency questionnaires-a review. Public Health Nutr.

[20] Masson LF, MCNeill G, Tomany J, Simpson J, Peace HS, Wei L (2003). Statistical approaches for assessing the relative validity of a food-frequency questionnaire: use of correlation coefficients and the kappa statistic. Public Health Nutr.

[21] Vittinghoff E, McCulloch CE (2007). Relaxing the rule of ten events per variable in logistic and Cox regression. Am J Epidemiol.

[22] Halpern G, Schor E, Kopelman A (2015). Nutritional aspects related to endometriosis. Rev Assoc Med Bras (1992).

[23] Harris HR, Eke AC, Chavarro JE, Missmer SA (2018). Fruit and vegetable consumption and risk of endometriosis. Hum Reprod.

[24] Longcope C, Feldman HA, McKinlay JB, Araujo AB (2000). Diet and sex hormone-binding globulin. J Clin Endocrinol Metab.

[25] Prentice R, Thompson D, Clifford C, Gorbach S, Goldin B, Byar D (1990). Dietary fat reduction and plasma estradiol concentration in healthy postmenopausal women.The Women's Health Trial Study Group. J Natl Cancer Inst.

[26] Adlercreutz H (1990). Western diet and Western diseases: some hormonal and biochemical mechanisms and associations. Scand J Clin Lab Invest Suppl.

[27] Bosetti C, Altieri A, La Vecchia C (2002). Diet and environmental carcinogenesis in breast/gynaecological cancers. Curr Opin Obstet Gynecol.

[28] Barnard ND, Scialli AR, Hurlock D, Bertron P (2000). Diet and sex-hormone binding globulin, dysmenorrhea, and premenstrual symptoms. Obstet Gynecol.

[29] Heilier JF, Donnez J, Nackers F, Rousseau R, Verougstraete V, Rosenkranz K (2007). Environmental and host-associated risk factors in endometriosis and deep endometriotic nodules: a matched case-control study. Environ Res.

[30] Samaneh Y, ShahidehJahanian S, Azadeh M, Anoshirvan K (2019). The association of food consumption and nutrient intake with endometriosis risk in Iranian women: A case-control study. Int J Reprod Biomed (Yazd).

[31] Sadeghi A, Sadeghian M, Nasiri M, Rahmani J, Khodadost M, Pirouzi A (2020). Carbohydrate quantity and quality affect the risk of endometrial cancer: A systematic review and dose-response metaanalysis. Clin Nutr.

[32] Fung TT, Schulze MB, Hu FB, Hankinson SE, Holmes MD (2012). A dietary pattern derived to correlate with estrogens and risk of postmenopausal breast cancer. Breast Cancer Res Treat.

[33] Andersson AM, Skakkebaek NE (1999). Exposure to exogenous estrogens in food: possible impact on human development and health. Eur J Endocrinol.

[34] Huhtinen K, Desai R, Ståhle M, Salminen A, Handelsman DJ, Perheentupa A (2012). Endometrial and endometriotic concentrations of estrone and estradiol are determined by local metabolism rather than circulating levels. J Clin Endocrinol Metab.

[35] Di Maso M, Talamini R, Bosetti C, Montella M, Zucchetto A, Libra M (2013). Red meat and cancer risk in a network of case-control studies focusing on cooking practices. Ann Oncol.

[36] Schulze W, Schultze-Petzold H, Hazem AS, Gross R (1978). Objectivization of pain and consciousness in the conventional (dart-gun anesthesia) as well as in ritual (kosher incision) slaughter of sheep and calf. Dtsch Tierarztl Wochenschr.

[37] Wolfler MM, Meinhold-Heerlein IM, Henkel C, Rath W, Neulen J, Maass N (2013). Reduced hemopexin levels in peritoneal fluid of patients with endometriosis. Fertil Steril.

[38] Harel Z, Biro FM, Kottenhahn RK, Rosenthal SL (1996). Supplementation with omega-3 polyunsaturated fatty acids in the management of dysmenorrhea in adolescents. Am J Obstet Gynecol.

[39] Zemel MB, Sun X (2008). Dietary calcium and dairy products modulate oxidative and inflammatory stress in mice and humans. J Nutr.

[40] Perez Rodrigo C, Aranceta J, Salvador G, Varela-Moreiras G (2015). Food frequency questionnaires. Nutr Hosp.

